# Clinical Outcome of FFR-Guided Revascularization Strategy of Coronary Lesions: The HALE-BOPP Study

**DOI:** 10.31083/j.rcm2402062

**Published:** 2023-02-14

**Authors:** Matteo Tebaldi, Francesco Gallo, Alessandra Scoccia, Alessandro Durante, Delio Tedeschi, Sebastiano Verdoliva, Bernardo Cortese, Ferruccio Bilotta, Stuart Watkins, Alfonso Ielasi, Giuliano Valentini, Rita Pavasini, Matteo Serenelli, Emanuele D’Aniello, Marco Arena, Graziella Pompei, Antonella Scala, Ennio Scollo, Federico Gibiino, Serena Caglioni, Daniela Mele, Andrea Marrone, Simone Biscaglia, Emanuele Barbato, Gianluca Campo

**Affiliations:** ^1^Cardiovascular Institute, Azienda Ospedaliera Universitaria S. Anna, 44124 Ferrara, Italy; ^2^Cardiology Department, Ospedale dell’Angelo di Mestre, 30174 Venice, Italy; ^3^Cardiology Department, Policlinico San Marco, 24040 Zingonia (BG), Italy; ^4^Cardiology Department, Istituto Clinico S. Anna, 25127 Brescia (BS), Italy; ^5^Cardiology Department, Clinica Montervergine, 83013 Mercogliano (AV), Italy; ^6^Cardiology Department, Clinica San Carlo, 20037 Paderno Dugnano (MI), Italy; ^7^Cardiology Department, Ospedale Civile SS Annunziata, 07100 Sassari (SS), Italy; ^8^Department of Interventional Cardiology, West of Scotland Regional Heart and Lung Centre, Golden Jubilee National Hospital, G81 4DY Glasgow, UK; ^9^Cardiology Department, Istituto Clinico Sant’Ambrogio, 20149 Milano (MI), Italy; ^10^Cardiology Department, Ospedale San Filippo e Nicola, 67051 Avezzano (AQ), Italy; ^11^Cardiology Department, Ospedale S. Andrea, 19121 La Spezia, Italy; ^12^Cardiovascular Center Aalst, OLV Clinic, 9300 Aalst, Belgium; ^13^Department of Advanced Biomedical Sciences, University Federico II, 80138 Naples, Italy

**Keywords:** fractional flow reserve, target vessel failure, FFR-based deferral, coronary revascularization

## Abstract

**Background:**

Recently, questions around the efficacy and effectiveness of Fractional Flow Reserve (FFR) have arisen in various clinical settings.

**Methods:**

The Clinical Outcome of FFR-guided Revascularization Strategy of Coronary Lesions (HALE-BOPP) study is an investigator-initiated, multicentre, international prospective study enrolling patients who underwent FFR measurement on at least one vessel. In accordance with the decision-making workflow and treatment, the vessels were classified in three subgroups: (i) angio-revascularized, (ii) FFR-revascularized, (iii) FFR-deferred. The primary endpoint was the occurrence of target vessel failure (TVF, cardiac death, target vessel myocardial infarction and ischemia-driven target vessel revascularization). The analysis was carried out at vessel- and patient-level.

**Results:**

1305 patients with 2422 diseased vessels fulfilled the criteria for the present analysis. Wire-related pitfalls and transient adenosine-related side effects occurred in 0.8% (95% CI: 0.4%–1.4%) and 3.3% (95% CI: 2.5%–4.3%) of cases, respectively. In FFR-deferred vessels, the overall incidence rate of TVF was 0.024 (95% CI: 0.019–0.031) lesion/year. After a median follow-up of 3.6 years, the occurrence of TVF was 6%, 7% and 11.7% in FFR-deferred, FFR-revascularized and angio-revascularized vessels, respectively. Compared to angio-revascularized vessels, FFR-guided vessels (both FFR-revascularized and FFR-deferred vessels) showed a lower TVF incidence rate lesion/year (0.029, 95% CI: 0.024–0.034 vs. 0.049, 95% CI: 0.040–0.061 respectively, *p* = 0.0001). The result was consistent after correction for confounding factors and across subgroups of clinical interest. The patient-level analysis confirmed the lower occurrence of TVF in negative-FFR vs. positive-FFR subgroups.

**Conclusions:**

In a large prospective observational study, an FFR-based strategy for the deferral of coronary lesions is a reliable and safe tool, associated with good outcomes.

**Clinical Trial Registration:**

NCT03079739.

## 1. Introduction

More than 20 years of research have supported the safety and effectiveness of a fractional flow reserve (FFR) guided coronary revascularization in different clinical settings, ranging from intermediate lesions in patients with chronic coronary syndrome (CCS) to non-culprit lesions in patients with acute coronary syndrome (ACS) [1, 2]. Nonetheless, the translation from randomized clinical trials (RCTs) to daily practice seems to have highlighted some pitfalls and concerns [3, 4]. In fact, some authors reported limitations related to lesion crossability, procedural time, costs, or adenosine side effects [5]. Others suggested that deferring lesions in a specific subset of patients (i.e., ACS, diabetic, chronic kidney disease, low ventricular ejection fraction, etc.) could be associated with a higher occurrence of adverse events [6]. Recent studies have questioned the advantage of an FFR-guided complete revascularization, reporting a similar outcome with the angio-guided approach [7, 8]. In agreement with this background, further evidence from real-life studies was needed to support the safety of FFR-guided deferral and the effectiveness of FFR-guided revascularization.

In the present analysis, the data of patients enrolled in a large multicentre prospective study were analyzed at vessel-level and patient-level to compare the long-term outcome of FFR-based deferrals vs. FFR-guided and angio-guided revascularization.

## 2. Materials and Methods

### 2.1 Study Design

**Fig. 1.**
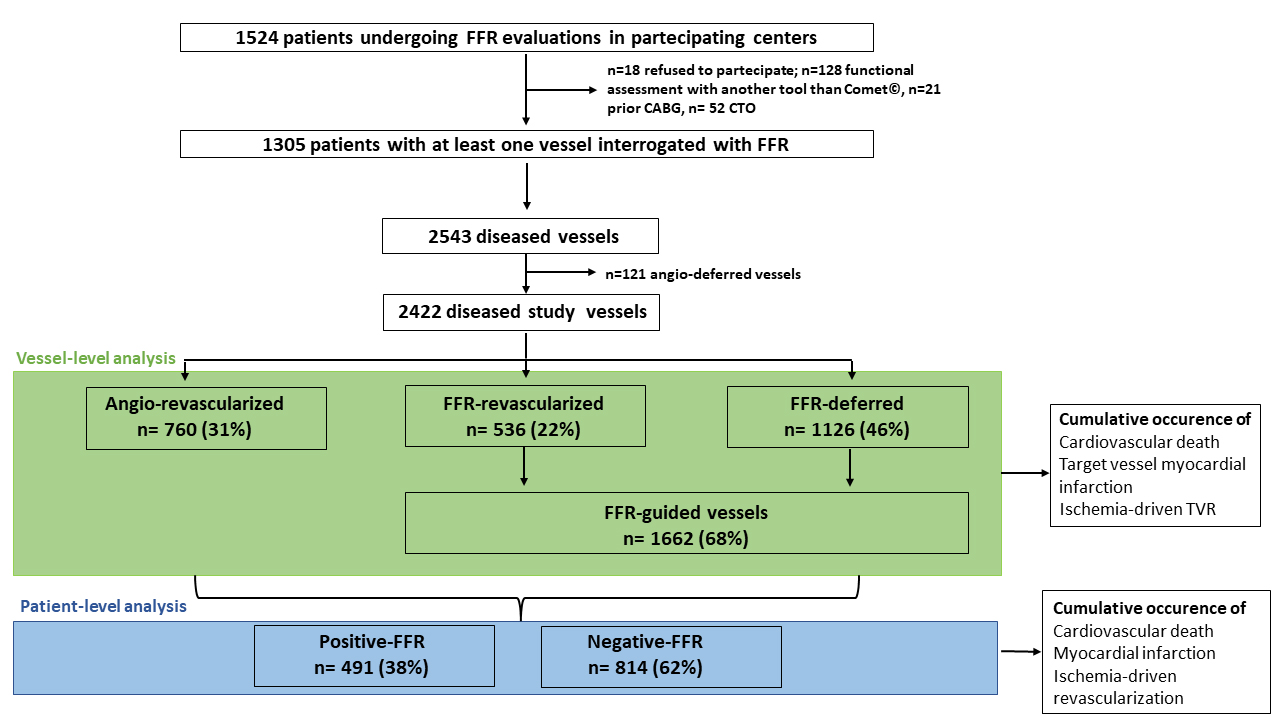
**Study flow-chart**. FFR, fractional flow reserve; CABG, coronary artery graft bypass; CTO, chronic total occlusion; TVR, target vessel revascularization.

The Clinical Outcome of FFR-guided Revascularization Strategy of Coronary Lesions (HALE-BOPP) study is an investigator-initiated, multicentre, international prospective study conducted in ten hospitals between Italy and the United Kingdom. The study organization and the participating centres are listed in the supplemental online. The study consecutively enrolled all patients who underwent FFR measurement on at least one vessel with COMET® wire (H74939359310, Boston Scientific, Natick, MA, USA) (Fig. 1). Exclusion criteria were life expectancy of less than one year because of known non-cardiovascular comorbidity, inability to guarantee clinical follow-up, and unwillingness to provide written informed consent. Patients with prior coronary artery bypass (CABG) and chronic total occlusion (CTO) were also excluded.

### 2.2 Study Procedures and Definitions

All vessels showing a lesion with a diameter stenosis (DS) ≥50% (by visual estimation) were of interest to the study. The final decision to measure FFR or to proceed with an angio-based revascularization or deferral was left to the operator. The protocol strongly suggested that vessels with the culprit lesion of ACS or with lesions showing DS ≥90% should be treated with percutaneous coronary intervention (PCI) and second-generation drug-eluting stent (DES) avoiding FFR measurement. For all the other vessels showing at least one lesion greater than 50%, the protocol strongly suggested performing an FFR evaluation to guide revascularization. An FFR assessment was performed according to the procedures previously described [9]. FFR requires the use of maximal hyperaemia, which can be induced by both systemic and intracoronary administration of adenosine. The FFR was calculated by evaluating the ratio between the proximal aortic pressure and distal coronary pressure during the steady state of maximal hyperaemia [10]. Potential pitfalls related to wire (i.e., drift, coronary dissection, coronary perforation, inability to cross the stenosis, etc.) were prospectively recorded. After each measurement, pressure-wire pullback to check for pressure-drift was strongly recommended. A drift-value from 0.96–1.04 was accepted. For FFR values of 0.76–0.84, a drift with a narrower range of 0.98–1.02 was accepted [11]. The FFR value was considered flow-limiting (positive) if ≤0.80 and coronary revascularization was mandated by protocol. Conversely, an FFR value >0.80 was considered not flow-limiting (negative), and coronary revascularization had to be deferred. In accordance with decision-making workflow and treatment, vessels were classified into three subgroups: (i) angio-revascularized, (ii) FFR-revascularized, (iii) FFR-deferred. A PCI procedure was performed using standard materials and techniques. Subsequently, patients received standard medical therapy according to the current guidelines [12], with particular emphasis on achieving the recommended targets of low-density lipoprotein cholesterol (LDL cholesterol). Clinical follow-up occurred at 1, 6, and 12 months and annually thereafter. For the present analysis, follow-up was censored in March 2022 or at the time of death.

### 2.3 Data Collection

All baseline, clinical, lesion, and outcome data were prospectively collected using a dedicated electronic case report form (eCRF). The specialized personnel at each centre followed this paradigm. Members of the academic coordinating centre (University of Ferrara, Ferrara, Italy) periodically performed monitoring and verification of data in the Italian hospitals. Members of the contract research organization GBPharma (Pavia, Italy) monitored and verified the data of the United Kingdom centre. Angiograms and FFR traces were prospectively analysed at an independent core laboratory (University of Ferrara, Ferrara, Italy) without knowledge of the patient’s outcomes. Angiographic analyses were carried out for all lesions with a 50% or greater DS in each epicardial vessel and side branch that were 1.5 mm or larger in diameter, using an automated edge-detection algorithm (QAngio XA 7.3, Medis Medical Imaging Systems, Leiden, Netherlands). FFR traces were reviewed for the quality in hyperaemia induction, drift check and consistency for the FFR value reported in the eCRF.

### 2.4 Outcomes

The main analysis was carried out at vessel-level. The study endpoint was the target vessel failure (TVF), defined as the cumulative occurrence of cardiac death, target vessel myocardial infarction and ischemia-driven target vessel revascularization. The prespecified time-point of the primary outcome was at 1 year. Due to the limited number of adverse events and to better describe the natural history of coronary lesions whose treatment was deferred based on the FFR results, the present analysis reports data outcome from the longest-term follow-up. Adverse events are defined in the supplemental online and were adjudicated by a clinical events committee that reviewed original source documents. In case of repeated adverse events, the first one that occurred was the one considered. In addition, the committee assigned each event to a specific coronary vessel based on the available information (i.e., electrocardiogram, cardiac biomarkers, echocardiography, coronary artery angiography). In the case of cardiovascular death in patients with multiple study vessels, the event was assigned to each vessel [13].

### 2.5 Additional Patient-Level Analysis

To support and confirm the findings of the vessel-level analysis and allow comparison with previous studies, data are also analysed at patient-level. According to the vessel status, patients were defined: (i) negative-FFR if all vessels interrogated with FFR were classified as FFR-deferred, (ii) positive-FFR if at least one interrogated vessel was classified as FFR-revascularized [6]. The composite study endpoint included cardiac death, myocardial infarction, and ischemia-driven coronary revascularization.

### 2.6 Statistical Analysis

Starting from previous similar studies [14, 15, 16], we expected a 1-year incidence of the endpoint at around 5% in the FFR-deferred vessels. Setting a tolerance margin at around 1.5%, at least 811 patients with at least one FFR-deferred vessel were needed. Continuous data were tested for normal distribution with the Kolmogorov-Smirnov test. Normally distributed values were presented as mean ± SD, otherwise, the median value and interquartile range (IQR) were used. Categorical variables were summarised in terms of counts and percentages. For the comparison between groups, *t*-test, Mann-Whitney U test and Pearson’s χ^2^ test were applied as appropriate. Unadjusted survival was examined with Kaplan-Meier survival curves and the log-rank test. Overall, we performed evaluations “per vessel”, but these evaluations were clustered by patient. In this way, the assumption of independence between vessels was violated. Therefore, to adjust for this clustering effect, we used multilevel modelling and shared frailty Cox proportional hazards regression as the primary model [17, 18]. Cox regression analysis with interaction testing was performed to determine whether the effect of the FFR-based deferral strategy (vs. coronary revascularization) on the primary endpoint was consistent across different subgroups of clinical interest. To support the findings of the vessel-level analysis, the analyses were also repeated at patient-level. All analyses were performed with Stata version 13.1 (StataCorp LP, College Station, TX, USA).

## 3. Results

From March 2017 to September 2019, 1305 patients fulfilled the criteria for the present analysis (enrolment at different sites was not simultaneous based on different regulatory approval timelines) (Fig. 1, Table 1). Overall, the number of vessels showing a lesion with DS ≥50% were 2543. In 121 (4.7%) vessels, even though it was discouraged by the protocol as they were not a culprit lesion of ACS and did not have a DS ≥90%, the treatment was deferred only based on the operator’s choice (angio-deferred vessels) (Fig. 1 and **Supplementary Table 1**). The remaining 2422 vessels (95.3%) were the study object (Table 2). The mean age was 68 years (Table 1). Around half of the patients were admitted for ACS, one quarter showed chronic kidney disease (CKD, as defined as baseline creatinine clearance <60 mL/min), and around 15% presented a value of left ventricular ejection fraction (LVEF) ≤40% (Table 1). The most frequent diseased vessel was the left anterior descending one (Table 2). More than 90% of cases were de novo lesions, and the proximal location of the coronary lesion was the most common (Table 2).

**Table 1.** **Study population**.

Patients (n = 1305)		
	Age, years	68.1 ± 10
	Female, no. (%)	354 (27.1)
	BMI, Kg/$m^{2}$	27.8 ± 4
Clinical history, no. (%)		
	Hypertension	1001 (76.7)
	Hyperlipidaemia	886 (68.6)
	Current smoking	239 (18.3)
	Diabetes mellitus	324 (24.8)
	Prior IHD	472 (36.2)
	Prior MI	320 (24.5)
	Prior PCI	422 (32.3)
	Prior CVA	60 (4.6)
	Peripheral artery disease	357 (27.4)
	COPD	71 (5.4)
	CKD	332 (25.4)
Clinical presentation		
	ACS, no. (%)	650 (49.8)
	STEMI	169 (12.9)
	NSTEMI	465 (35.6)
	UA	16 (1.2)
	CCS, no. (%)	655 (50.2)
	Stress test done, no. (%)	248 (43.9)
	Imaging stress test, no. (%)	88 (35.5)
	Positive stress test, no. (%)	226 (91.1)
	LVEF, %	53.2 ± 9
	LVEF <40%, no. (%)	180 (13.8)
	Multivessel disease, no. (%)	899 (68.9)
	Multivessel revascularization, no. (%)	354 (27.1)
Discharge medication, no. (%)		
	Aspirin	1280 (98.1)
	P2Y12 inhibitors	1243 (95.2)
	Oral anticoagulants	20 (1.5)
	ACE inhibitors or ARB	1191 (91.2)
	Beta blockers	1128 (86.4)
	Statin	1189 (91.1)
	high-dose statin	892 (75.0)
	Ezetimibe	203 (15.5)

BMI, body mass index; IHD, ischemic heart disease; MI, myocardial infarction; PCI, percutaneous coronary intervention; CVA, cerebrovascular accident; COPD, chronic obstructive pulmonary disease; CKD, chronic kidney disease; ACS, acute coronary syndrome; STEMI, ST-segment elevation MI; NSTEMI, non-ST-segment elevation MI; UA, unstable angina; CCS, chronic coronary syndrome; LVEF, left ventricular ejection fraction; ACE, angiotensin converting enzyme; ARB, angiotensin 2 receptor blocker.

**Table 2.** **Vessel characteristics across the three study groups**.

		Angio-revascularized	FFR-revascularized	FFR-deferred	*p*
		(n = 760)	(n = 536)	(n = 1126)	
Territory, no. (%)					<0.001
	Left main	24 (3.2)	29 (5.4)	36 (3.2)	
	LAD	192 (25.3)	396 (73.9)	524 (46.5)	
	LCx	244 (32.1)	58 (10.8)	324 (28.8)	
	RCA	300 (39.5)	53 (9.9)	242 821.5)	
Lesion features					
	Type, no. (%)				<0.001
	De novo	717 (94.3)	479 (89.4)	1054 (93.6)	
	In-stent restenosis	39 (5.1)	57 (10.6)	71 (6.3)	
	Other	4 (0.5)	0 (0)	1 (0.1)	
	Serial lesions, no. (%)	99 (13.0)	122 (22.8)	132 (11.7)	<0.001
	Location, no. (%)				<0.001
	Proximal	323 (42.5)	355 (66.2)	616 (54.7)	
	Mid	172 (22.6)	136 (25.4)	321 (28.5)	
	Distal	265 (34.9)	45 (8.4)	189 (16.8)	
	AHA/ACC classification, no. (%)				<0.001
	A or B1	208 (27.4)	112 (20.9)	530 (47.1)	
	B2	289 (38.0)	287 (53.6)	500 (44.4)	
	C	263 (34.6)	134 (25.0)	89 (7.9)	
	Severe calcification, no. (%)	122 (16.1)	86 (16.0)	97 (8.6)	<0.001
	Bifurcation, no. (%)	234 (30.8)	222 (41.4)	345 (30.6)	<0.001
	Severe tortuosity, no. (%)	21 (2.8)	21 (3.9)	54 (4.8)	0.085
Quantitative coronary analysis					
	RVD, mm	2.57 ± 1.18	2.55 ± 0.61	2.72 ± 0.69	0.001
	Diameter stenosis, %	66.43 ± 18.11	58.46 ± 10.22	56.83 ± 9.91	<0.001
	Lesion length, mm	14.71 ± 12.38	15.05 ± 12.17	12.23 ± 8.70	<0.001
	MLD, mm	1.12 ± 1.11	1.24 ± 2.03	1.36 ± 1.70	0.053

LAD, left anterior descending; LCx, left circumflex; RCA, right coronary artery; AHA, American Heart Association; ACC, American College of Cardiology; RVD, reference vessel diameter; MLD, minimal lumen diameter; A, B1, B2 and C are parts of classification, is not an abbreviation.

### 3.1 Decision-Making Workflow and FFR Measurement

Operators proceeded with angio-guided revascularization in 760 vessels (angio-revascularized, Table 2, **Supplementary Table 2**). The reasons behind the use of the angio-guided approach as reported by the operators are recorded in **Supplementary Table 3**. Conversely, in 1662 vessels, the FFR was assessed to guide revascularization (FFR-guided vessels, Table 2). Overall, in 1126 (67%) vessels, the treatment was deferred (FFR-deferred), whereas 536 (33%) vessels were treated with PCI (FFR-revascularized) (Table 2). During the FFR assessment, 14 (0.8%) cases of wire-related pitfalls were observed (drift n = 12, inability to cross the lesion n = 2). No perforations or dissections were reported. Adenosine was administered intravenously in 1459 (87.9%) measurements. Fifty-six (3.3%) patients complained adenosine-related side effects (significant dyspnoea n = 51, hypotension n = 3, marked bradycardia n = 2). The median FFR value in the FFR-revascularized group was 0.74 [0.70–0.78], while it was 0.88 [0.84–0.92] in the FFR-deferred group.

In order to better characterise the vessels under consideration, the characteristics and annual event rate of the 121 vessels that were deferred on the basis of the angiographic evaluation (Angio-deferred) are also reported in the online **Supplementary Table 1**.

### 3.2 FFR-Deferred Vessels-Primary Outcome

At 1-year, 13 (1.1%) cardiovascular deaths, 9 (0.6%) target vessel myocardial infarction (MI), and 15 (1.3%) ischemia-driven target vessel revascularizations occurred in the FFR-deferred vessels. TVF occurred in 29 (2.5%, 95% CI: 1.9%–3.1%) vessels, which was significantly inferior to the prespecified primary endpoint estimation (from 3.5% to 6.5%). During a median follow-up of 3.6 [2.5–4.7] years, 31 (2.8%) cardiovascular deaths, 19 (1.7%) target vessel MI and 38 (3.4%) ischemia-driven target vessel revascularizations occurred. Altogether, TVF occurred in 68 (6%) vessels. The overall incidence rate of TVF was 0.024 (95% CI: 0.019–0.031) lesion/year. The univariate associations between potential predictor covariates and TVF are shown in** Supplementary Table 4**. The final predictors of TVF in FFR-deferred vessels were CKD (hazard ratio [HR]: 2.74, 95% CI: 1.22–6.12), multivessel disease (HR: 3.69, 95% CI: 1.22–6.12) and LVEF (0.95, 95% CI: 0.92–0.98).

### 3.3 FFR-Revascularized – Primary Outcome 

At the longest-term follow-up, the TVF incidence rate lesion/year and the unadjusted TVF cumulative occurrence in the FFR-revascularized vessels were 0.029 (95% CI: 0.02–0.03) and 7%, respectively. They were significantly lower than those of the angio-revascularized vessels (0.049, 95% CI: 0.040–0.061, *p *= 0.0001 and 11.7%, HR: 0.58, 95% CI: 0.44–0.76, *p *= 0.0001, respectively).

### 3.4 Additional Analyses – Primary Outcome

**Fig. 2.**
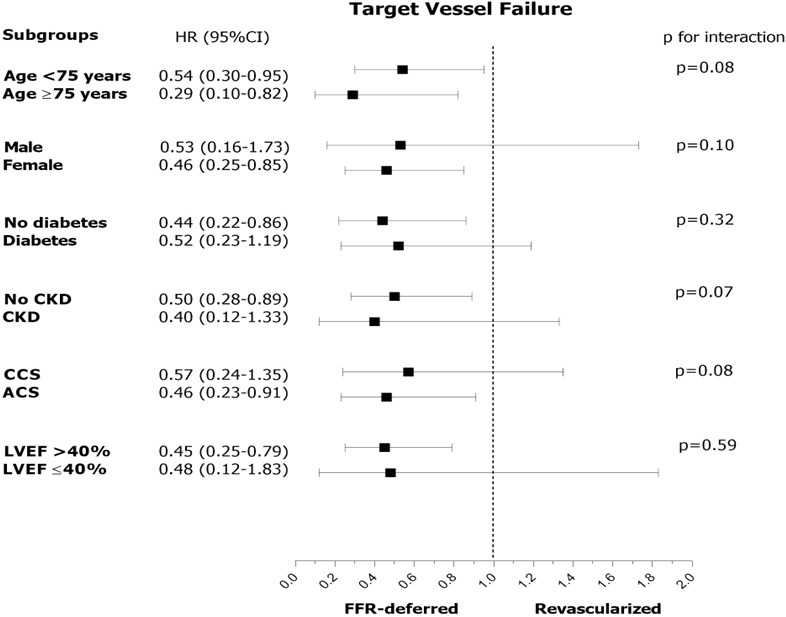
**Subgroup analysis in the comparison of FFR-deferral vs. revascularization**. HR, hazard risk; CKD, chronic kidney disease; CCS, chronic coronary syndrome; ACS, acute coronary syndrome; LVEF, left ventricular ejection fraction; LM, left main; LAD, left anterior descending artery; RCA, right coronary artery; LCx, left circumflex artery; FFR, fractional flow reserve.

**Fig. 3.**
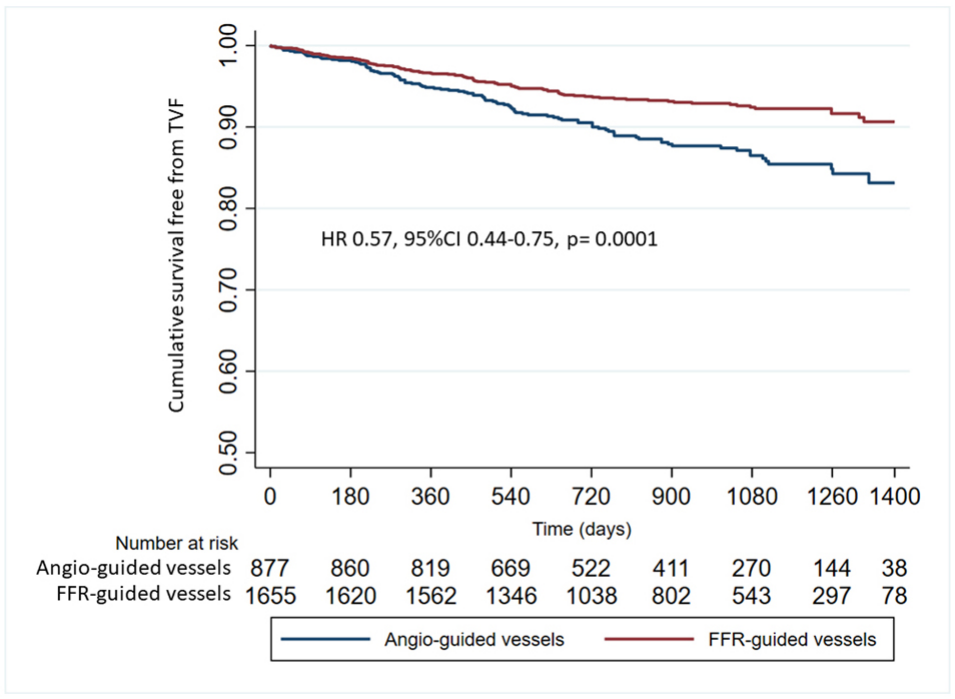
**Cumulative survival free from target vessel failure in FFR-guided vessels vs. Angio-guided vessels**. FFR, fractional flow reserve; HR, hazard risk.

TVF occurred in 15 (12.4%) angio-deferred vessels. The incidence rate of TVF was 0.055 (95% CI: 0.033–0.092). As compared to angio-deferral, FFR-deferral was an independent protective factor for TVF also after correction for potential patient’s and vessel’s confounding factors (HR: 0.38, 95% CI: 0.18–0.79, *p* = 0.01). Similarly, compared to revascularized vessels (angio- and FFR-revascularized, target vessel revascularization [TVR] incidence rate lesion/year 0.040, 95% CI: 0.033–0.047, unadjusted TVF cumulative occurrence 9.4%), FFR-deferral was independently associated with a better outcome also after correction for potential patient’s and vessel’s confounding factors (HR: 0.69, 95% CI: 0.48–0.98, *p *= 0.044). This observation was consistent across subgroups of clinical interest (Fig. 2). Compared to angio-guidance (angio-deferred + angio-revascularized vessels), FFR-guidance (FFR-deferred + FFR-revascularized vessels) was associated with a lower occurrence of TVR (Fig. 3) and with a significant reduction of risk of TVF (group HR: 0.58, 95% CI: 0.44–0.76, *p *= 0.0001). The predictors of TVF in all study vessels were female sex (0.41, 95% CI: 0.20–0.84, *p* = 0.014), CKD (2.15, 95% CI: 1.16–3.96, *p *= 0.014), ACS (2.75, 95% CI: 1.55–4.86, *p* = 0.0001), de novo lesion (0.37, 95% CI: 0.17–0.80, *p *= 0.012) (**Supplementary Table 5**).

### 3.5 Patient-Level Analysis-Primary Outcome 

Overall, 814 (62%) and 491 (38%) patients were classified as negative-FFR and positive-FFR, respectively (Fig. 1). At the longest available follow-up, 40 (3.0%) patients died of a cardiovascular cause, 63 (4.8%) experienced MI and 110 (8.4%) underwent ischemia-driven coronary revascularization. The TVF incidence rate lesion/year was significantly lower in the negative-FFR patients compared to the positive-FFR ones (0.027, 95% CI: 0.020–0.035 vs. 0.044, 95% CI: 0.033–0.058, *p *= 0.021). Also, the unadjusted TVF cumulative occurrence was lower in negative-FFR patients (HR: 0.63, 95% CI: 0.42–0.93, *p* = 0.021) (**Supplementary Fig. 1**). After correction for potential confounding factors, female sex (HR: 0.51, 95% CI: 0.28–0.91), diabetes mellitus (HR: 0.49, 95% CI: 0.26–0.91), LVEF (HR: 0.96, 95% CI: 0.94–0.99) and multivessel disease (HR: 4.71, 95% CI: 2.37–9.59) were independent predictors of TVF (**Supplementary Table 6**). We found a significant interaction between ACS and CCS patients (0.79, 95% CI: 0.53–1.16 vs. 0.75, 95% CI: 0.40–1.39, *p* for interaction = 0.007) and between patients with or without diabetes (0.32, 95% CI: 0.15–0.68 vs. 0.99, 95% CI: 0.68–1.44, *p* for interaction = 0.017) (**Supplementary Fig. 2**).

## 4. Discussion

The HALE BOPP study was conducted to investigate the long-term outcome of FFR-guided revascularization strategies for coronary lesions. The HALE BOPP study collected data from consecutive patients undergoing coronary revascularization with contemporary techniques and devices, receiving intracoronary physiology with a standardized approach using the same tool, and treated with optimal medical therapy and updated secondary prevention. This study has two major strengths. First, the inclusion of a real-life study population, including around 50% of patients admitted to hospital for ACS, with complex coronary anatomy where coronary physiology is systematically applied to guide coronary revascularization. Second, the adverse events are centrally adjudicated and attributed to the responsible vessel. This allowed both vessel-level and patient-level analyses, but also the possibility to discriminate the adverse events related to either coronary physiology, revascularization, or deferral. The main findings are as follows:

(i) The extensive use of coronary physiology with contemporary tools in daily practice is feasible and related to a very low rate of minor issues (wire-related pitfalls 0.8%, 95% CI: 0.4%–1.4%, transient adenosine-related side effects 3.3%, 95% CI: 2.5%–4.3%).

(ii) An FFR-based deferral strategy is related to a reasonable and acceptable number of adverse events (2.5%, 95% CI: 1.9%–3.1% at 1-year and 6.0%, 95% CI: 4.3%–7.1% at a median of 3.5 years follow-up), consistent across several clinical subgroups.

(iii) Multivessel disease, CKD and LVEF were associated with a higher occurrence of adverse events in the FFR-deferred vessels.

(iv) Compared to angio-revascularized vessels, FFR-guided vessels (both FFR-revascularized and FFR-deferred vessels) showed a lower TVF incidence rate lesion/year, a finding that was consistent after correction for confounding factors and across subgroups of clinical interest.

Currently, the use of coronary physiology across countries, laboratories, and operators significantly differs, and is still relatively low, despite being a class I indication in American and European guidelines [3, 19]. Several reasons have been put forward to explain this phenomenon, and several solutions have been proposed. To overcome adenosine-related side effects, resting indexes have been developed [20]. To minimize time and technical constraints, better performing wires, alternative tools (i.e., microcatheter for FFR measurement and/or angio-derived FFR) and more user-friendly interfaces have been produced [21, 22, 23, 24]. Nonetheless, the major barrier remains the operators’ skepticism regarding the deferral of coronary lesions, the risk of adverse events related to the untreated lesions (especially in some specific high-risk subsets of patients, i.e., those admitted for ACS), and the seemingly limited benefit compared to an angio-based approach.

Approximately 50% of the population of the study consists of patients with ACS. In this presentation setting, the use of functional assessment could be debated for two main reasons: (i) the presence of coronary microcirculation dysfunction related to the acute event could lead to an underestimation of the FFR value; (ii) the inability to identify the true culprit lesion may lead to performing a functional analysis on a vessel in which an assessment with FFR is conceptually incorrect and may lead to misinterpretation of stenosis. Recent papers have further fuelled the debate. Cerrato *et al*. [6] reported a higher rate of adverse events in the deferred ACS group, compared to the deferred CCS group. This difference was not present in revascularized patients. However, it should be noted that the analysis was carried out at patient-level, and it is unclear whether the adverse events could be attributed to the deferred vessel. In fact, in our data, we noted an increase in events in ACS patients if we performed the analysis at patient level. However, at vessel level, this finding is not confirmed. This could confirm the hypothesis whereby the increase in events is linked to the complexity of the ACS patient rather than the failure of functional assessment in the patient setting. The Flow Evaluation to Guide Revascularization in Multivessel ST-Elevation Myocardial Infarction (FLOWER-MI) trial did not find significant benefits of the FFR-guided complete revascularization over the angio-guided one [8]. However, the study included a highly selected population, and the functional evaluation was performed in a staged procedure in the majority of patients (despite the protocol suggesting the opposite), hence limiting the potential FFR advantage in reducing the number of unnecessary procedures. That is why the observed rate of adverse events was significantly lower than expected.

Compared to these observations, the data from HALE BOPP are reassuring, confirming and adding to previous evidence from large real-life registries. We found that modern pressure wires can guarantee a high performance with a low number of complications. The cases where it was not possible to cross the lesion and perform FFR measurements are irrelevant in number, similarly to those with unacceptable drift requiring repeating the assessment. A systematic review reported a wide variability of device failures based on the tools (wires or microcatheter) and study population, ranging from 2% to 7% [25]. We reported a device failure rate of <1%.

Similarly, the occurrence of adenosine-side effects is far below the reported values of around 30% in other studies [26, 27]. We acknowledge that the way the study was organized might have generated results focusing on the more evident symptoms and issues. Nonetheless, the latter should be considered clinically meaningful and relevant for daily practice.

The FFR’s ability to discriminate coronary lesions requiring PCI is well-established and validated over time in different clinical settings. What is more relevant, is the rate of adverse events of FFR-deferred vessels, which is in line with the expectation and the natural history of atherosclerotic disease [28, 29]. After a median follow-up of 3.5 years, the cumulative occurrence of TVF in the FFR-guided vessels was 7%. This rate was significantly lower than that observed in the angio-revascularized vessels (11.7%, *p* = 0.001). This observation was confirmed after correcting several clinical and lesion characteristics in the main clinically meaningful subsets of patients. We did not find a significant interaction between subgroups stratified according to clinical presentation, age, sex, ventricular dysfunction, etc. Interestingly, we found that CKD, LVEF and multivessel disease were associated to an increased risk of developing adverse events in FFR-deferred vessels, risk factors already highlighted in previous studies [3, 30]. Finally, although the major strength of the present study is the vessel-level analysis, we also performed a patient-level analysis to test the consistency of our data and to allow comparison with previous studies. This analysis confirmed the overall satisfactory outcome of negative-FFR patients. These patients received fewer revascularizations, fewer stents and the long-term outcome was characterized by few adverse events imputable to FFR-deferred lesions.

## 5. Study Limitations

The present analysis is based on an observational study, then subjected to all potential limitations of these typologies of studies. We cannot exclude potential unmeasured confounding factors related to the operator’s decision to perform FFR or not in some vessels and to proceed with angio-based PCI in others. Data were collected in a limited number of centres (n = 10) and countries (n = 2), and their transferability should be further confirmed. In the vessel-level analysis, cardiovascular death is associated with all vessels increasing the overall number of events. However, this methodology is well-established and validated, and the findings from the ancillary patient-level analysis are consistent.

## 6. Conclusions

**Fig. 4.**
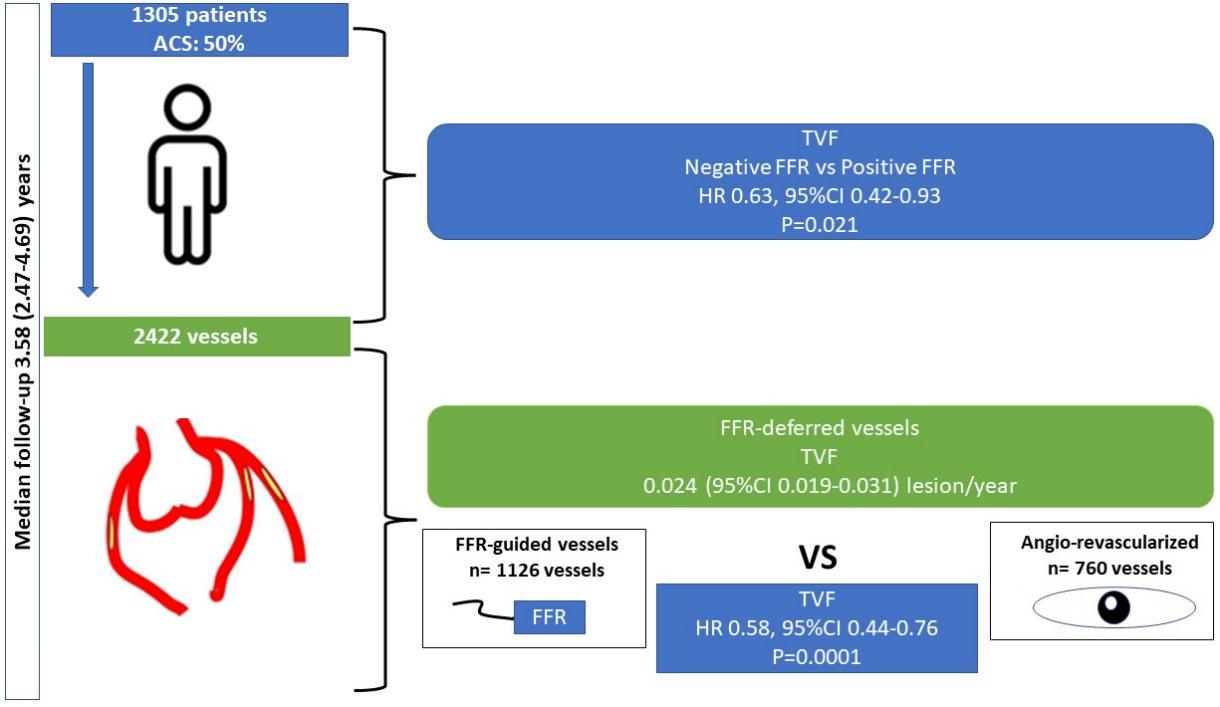
**Central Illustration**.

In a large prospective observational study, the FFR-based strategy for the deferral of coronary lesions is reliable, safe, and associated with a good clinical outcome (Fig. 4).

## Data Availability

The datasets used and/or analyzed during the current study are available from the corresponding author on reasonable request.

## Supplementary Material

Supplementary material associated with this article can be found, in the online version, at https://doi.org/10.31083/j.rcm2402062.
