# Supplementary material for: Clinical Outcome of FFR-Guided Revascularization Strategy of Coronary Lesions: The HALE-BOPP Study
Source: Rev Cardiovasc Med. 2023 Feb 14;24(2):62. doi: 10.31083/j.rcm2402062 (PMC11273154; doi:10.31083/j.rcm2402062)
Supplement: Supplementary file 1 [file 2153-8174-24-2-062-s1.zip › 2153-8174-24-2-062-s1.docx]

**Clinical Outcome of FFR-guided Revascularization Strategy of Coronary Lesions: the HALE-BOPP Study**

**Supplemental Material**

# Study Organization

- **Executive Committee**

Matteo Tebaldi (Ferrara), Alessandro Durante (Zingonia), Delio Tedeschi (Brescia), Stuart Watkins (Glasgow), Gianluca Campo (Ferrara).

- **Statistics committee**

Matteo Serenelli (Ferrara), Elisa Maietti (Bologna)

- **Data management**

Chiara Manzalini (Ferrara), Elisa Mosele (Ferrara), Martina Viola (Ferrara), Veronica Lodolini (Ferrara), Sara Papetti (Pavia)

- **Angiography and Physiology Core laboratory**

Carlo Tumscitz (Ferrara), Simone Biscaglia (Ferrara), Andrea Erriquez (Ferrara)

- **Clinical Event Committee**

Rita Pavasini (Ferrara), Paolo Cimaglia (Cotignola)

- **Participating centers and Investigators**
- Azienda Ospedaliera Universitaria di Ferrara, Cona (FE), Italy: Gianluca Campo, Carlo Tumscitz, Carlo Penzo, Matteo Tebaldi, Simone Biscaglia, Andrea Erriquez
- Department of Interventional Cardiology, West of Scotland Regional Heart and Lung Centre, Golden Jubilee National Hospital, Glasgow, United Kingdom.: Stuart Watkins
- Clinica Montervergine, Mercogliano (AV), Italy: Tullio Tesorio, Luigi Salemme, Angelo Cioppa, Sebastiano Verdoliva
- Ospedale San Filippo e Nicola, Avezzano (AQ), Italy: Giuliano Valentini
- Ospedale Valduce, Como (CO), Italy: Alessandro Durante
- Istituto Clinico S. Anna, Brescia (BS), Italy: Delio Tedeschi, Andrea Rizzi
- Clinica San Carlo, Paderno Dugnano (MI), Italy: Bernardo Cortese, Elena Viganò
- Ospedale Civile SS Annunziata, Sassari (SS), Italy: Ferruccio Bilotta, Guido Parodi
- Istituto Clinico Sant’Ambrogio, Milano (MI): Alfonso Ielasi, Maurizio Tespili
- **Sponsor**
- Azienda Ospedaliera Universitaria di Ferrara

The HALE-BOPP study received an unconditioned economic support by Boston Scientific. Boston Scientific had no role in the collection, analysis, and interpretation of data. Boston Scientific had no access to database and the responsibility of analyzing the data, writing the report and the decision to submit the paper for publication is solely of the Investigators.

# Definition of endpoints

- **Cardiovascular death**

Deaths will be classified as cardiovascular, non-cardiovascular or undetermined cause according to ARC-2 criteria. Deaths of undetermined cause will default to cardiovascular. Deaths related to the procedure or complications of the procedure (or concomitant treatment) will be classified as cardiovascular. Only deaths due to a documented non-cardiovascular cause (e.g., cancer) will be classified as non-cardiovascular.

- **Myocardial infarction**

MI will be defined according to the 4th universal definition of myocardial infarction. In particular, the term acute myocardial infarction should be used when there is an acute myocardial injury with clinical evidence of acute myocardial ischemia and with detection of a rise and/or fall of cardiac troponin values with at least one value above the 99^th^ percentile URL and at least one of the following: i) symptoms of myocardial ischemia, ii) new ischemic ECG changes, iii) development of pathological Q waves, iv) imaging evidence of new loss of viable myocardium or new regional wall motion abnormality in a pattern of consistent with ischemic aetiology, v) identification of a coronary thrombus by angiography or autopsy.

- **Ischemia-driven revascularization**

Any coronary revascularization (percutaneous or surgical) is defined as ischemia-driven if it respects the following criteria:

1. Ischemic symptoms consistent with CCS class ≥ 3 angina despite optimal medical therapy

AND

2. PCI or CABG of the study vessel

PLUS at least ONE of the following:

A. Positive functional study (exercise or myocardial perfusion imaging or stress or dobutamine echo) demonstrating clear evidence of reversible ischemia corresponding to a stenosis in the study vessel

B. New ischemic ECG changes (ST-segment depression ≥ 1 mm, ST-segment elevation ≥ 1 mm or T wave inversion ≥ 2 mm) at rest or with exertion in a distribution consistent with stenosis in a study vessel

C. Positive intracoronary physiology in a lesion in a study vessel.

Supplemental Table 1. Main characteristics of angio-deferred vessels.

|  | **Angio-deferred (n = 121)** |
| --- | --- |
|  |  |
| **Territory, no. (%)** |  |
| Left main | 4 (3.3) |
| LAD | 7 (5.8) |
| LCx | 41 (33.9) |
| RCA | 69 (57.0) |
| **Lesion features** |  |
| Type, no. (%) |  |
| De novo | 112 (92.6) |
| Instent restenosis | 8 (6.6) |
| Other | 1 (0.8) |
| Serial lesions, no. (%) | 11 (9.1) |
| Location, no. (%) |  |
| Proximal | 47 (38.8) |
| Mid | 31 (25.6) |
| Distal | 43 (35.5) |
| AHA/ACC classification, no. (%) |  |
| A or B1 | 36 (29.8) |
| B2 | 29 (24.0) |
| C | 56 (46.2) |
| Severe calcification, no. (%) | 31 (25.6) |
| Bifurcation, no. (%) | 17 (14.0) |
| Severe tortuosity, no. (%) | 7 (5.8) |
| **Quantitative coronary analysis** |  |
| RVD, mm | 2.46 ± 0.61 |
| Diameter stenosis, % | 70.9 ± 20 |
| Lesion length, mm | 18.9 ± 8 |
| MLD, mm | 0.73 ± 0.8 |

LAD, left anterior descending; LCx, left circumflex; RCA, right coronary artery; AHA, American Heart Association; ACC, American College of Cardiology; RVD, reference vessel diameter; MLD, minimal lumen diameter.

Supplemental Table 2. Main characteristics of study vessels.

|  | **All Vessels (n=2422)** | **FFR guided vessels (n= 1662)** | **Angio-revascularized vessels (n=760)** | ***p*** |
| --- | --- | --- | --- | --- |
|  |  |  |  |  |
| **Territory, no. (%)** |  |  |  | <0.001 |
| Left main | 89 (3.7) | 65 (3.9) | 24 (3.2) |  |
| LAD | 1112 (45.9) | 920 (55.4) | 192 (25.3) |  |
| LCx | 626 (25.8) | 382 (23.0) | 244 (32.1) |  |
| RCA | 595 (24.6) | 295 (17.7) | 300 (39.5) |  |
| **Lesion features** |  |  |  |  |
| Type, no. (%) |  |  |  |  |
| De novo | 2250(92.9) | 1533 (92.2) | 717 (94.3) | 0.005 |
| Instent restenosis | 167 (6.9) | 128 (7.7) | 39 (5.1) |  |
| Other | 5 (0.2) | 1 (0.1) | 4 (0.5) |  |
| Serial lesions, no. (%) | 353 (14.6) | 254 (15.3) | 99 (13.0) | 0.14 |
| Location, no. (%) |  |  |  | <0.001 |
| Proximal | 1294 (53.4) | 971 (58.4) | 323 (425.5) |  |
| Mid | 629 (26.0) | 457 (27.5) | 172 (22.6) |  |
| Distal | 499 (20.6) | 234 (14.1) | 265 (34.9) |  |
| AHA/ACC classification, no. (%) |  |  |  | <0.001 |
| A or B1 | 850 (35.1) | 642 (38.6) | 208 (27.4) |  |
| B2 | 1076 (44.4) | 780 (46.9) | 287 (37.8) |  |
| C | 486 (20.1) | 240 (14.4) | 265 (34.8) |  |
| Severe calcification, no. (%) | 3065 (12.6) | 183 (11.0) | 122 (16.1) | <0.001 |
| Bifurcation, no. (%) | 801 (33.1) | 567 (34.1) | 234 (30.8) | 0.11 |
| Severe tortuosity, no. (%) | 96 (4.0) | 75 (4.5) | 21 (2.8) | 0.041 |
| **Quantitative coronary analysis** |  |  |  |  |
| RVD, mm | 2.64 ± 0.84 | 2.67 ± 0.68 | 2.57 ± 1.18 | 0.041 |
| Diameter stenosis, % | 59.98 ± 13.56 | 57.32 ± 10.03 | 66.43 ± 18.11 | <0.001 |
| Lesion length, mm | 13.51 ± 10.66 | 13.08 ± 9.96 | 14.71 ± 12.38 | 0.010 |
| MLD, mm | 1.27 ± 1.65 | 1.32 ± 1.80 | 1.12 ± 1.11 | 0.032 |

LAD, left anterior descending; LCx, left circumflex; RCA, right coronary artery; AHA, American Heart Association; ACC, American College of Cardiology; RVD, reference vessel diameter; MLD, minimal lumen diameter.

Supplemental Table 3. Reasons for not performing functional assessment.

|  | **Angio-revascularized (N = 760)** |
| --- | --- |
| **Clinical decision, no. (%)** |  |
| ACS culprit lesion | 650 (85) |
| Co-culprit lesion in a patient with ACS | 20 (2.6) |
| DS ≥90% (significant lesion) | 50 (6.5) |
| FFR does not improve my ability to stratify lesions (significant lesion) | 30 (4) |
| Severe tortuosity | 10 (1.5) |

Supplemental Table 4. Univariate and multivariable analyses for TVF in FFR-deferred vessels

|  |  | **Univariate** | | | **Multivariable** | | |
| --- | --- | --- | --- | --- | --- | --- | --- |
|  |  | **HR** | **95%CI** | **p** | **HR** | **95%CI** | ***p*** |
| Age | SCU | 1.02 | 0.99–1.06 | 0.13 |  |  |  |
| Female | yes vs. no | 0.39 | 0.16–0.99 | 0.003 |  |  |  |
| BMI, Kg/m^2^ | SCU | 0.97 | 0.88–1.07 | 0.58 |  |  |  |
| Hypertension | yes vs. no | 2.69 | 0.94–7.67 | 0.064 |  |  |  |
| Hyperlipidemia | yes vs. no | 1.96 | 0.79–4.89 | 0.14 |  |  |  |
| Current smoking | yes vs. no | 0.95 | 0.55–1.61 | 0.85 |  |  |  |
| Diabetes mellitus | yes vs. no | 0.61 | 0.23–1.62 | 0.33 |  |  |  |
| Prior MI | yes vs. no | 1.40 | 0.57–3.41 | 0.45 |  |  |  |
| Prior PCI | yes vs. no | 1.03 | 0.44–2.41 | 0.93 |  |  |  |
| Prior CVA | yes vs. no | 4.07 | 0.81–20.44 | 0.08 |  |  |  |
| Peripheral artery disease | yes vs. no | 1.48 | 0.59–3.69 | 0.39 |  |  |  |
| COPD | yes vs. no | 2.72 | 0.54–13.73 | 0.22 |  |  |  |
| CKD | yes vs. no | 3.21 | 1.33–7.74 | 0.009 | 2.74 | 1.22–6.12 | 0.014 |
| ACS | yes vs. no | 2.64 | 1.19–5.83 | 0.016 |  |  |  |
| LVEF | SCU | 0.94 | 0.91–0.98 | 0.004 | 0.95 | 0.92–0.98 | 0.011 |
| Multivessel disease | yes vs. no | 4.45 | 1.67–11.80 | 0.003 | 3.69 | 1.41–9.63 | 0.008 |
| Aspirin | yes vs. no | 0.99 | 0.77–1.53 | 0.678 |  |  |  |
| P2Y12 inhibitors | yes vs. no | 0.96 | 0.54–1.65 | 0.832 |  |  |  |
| Oral anticoagulants | yes vs. no | 1.34 | 0.78–3.21 | 0.523 |  |  |  |
| ACE inhibitors or ARB | yes vs. no | 1.15 | 0.80–1.69 | 0.425 |  |  |  |
| Beta blockers | yes vs. no | 0.99 | 0.67–2.21 | 0.719 |  |  |  |
| Statin | yes vs. no | 0.96 | 0.59–2.01 | 0.321 |  |  |  |
| Ezetimibe | yes vs. no | 0.99 | 0.77–1.53 | 0.678 |  |  |  |
| Territory | LM/LAD vs. other | 0.67 | 0.35–1.29 | 0.23 |  |  |  |
| Type of lesion | De Novo vs. other | 0.76 | 0.21–2.69 | 0.67 |  |  |  |
| Serial lesions | yes vs. no | 0.87 | 0.28–2.69 | 0.81 |  |  |  |
| Location | proximal vs. other | 1.67 | 0.83–3.36 | 0.15 |  |  |  |
| AHA/ACC classification | A/B1 vs. B2/C | 0.43 | 0.21–0.90 | 0.027 |  |  |  |
| Severe calcifications | yes vs. no | 1.22 | 0.31–4.67 | 0.76 |  |  |  |
| Severe tortuosity | yes vs. no | 1.11 | 0.82–1.70 | 0.68 |  |  |  |
| RVD | SCU | 1.32 | 0.76–2.28 | 0.31 |  |  |  |
| Diameter stenosis | SCU | 1.01 | 0.97–1.06 | 0.43 |  |  |  |
| Lesion length | SCU | 0.95 | 0.90–1.00 | 0.079 |  |  |  |
| MLD | SCU | 0.82 | 0.45–1.49 | 0.52 |  |  |  |

SCU, single change unit; BMI, body mass index; MI, myocardial infarction; PCI, percutaneous coronary intervention; CVA, cerebrovascular accident; COPD, chronic obstructive pulmonary disease; CKD, chronic kidney disease; ACS, acute coronary syndrome; LVEF, left ventricular ejection fraction; ACE, angiotensin converting enzyme; ARB, angiotensin 2 receptor blocker; AHA, American Heart Association; ACC, American College of Cardiology; RVD, reference vessel diameter; FFR, fractional flow reserve.

Supplemental Table 5. Univariate and multivariable analyses for TVF in study vessels.

|  |  | **Univariate** | | | **Multivariable** | | |
| --- | --- | --- | --- | --- | --- | --- | --- |
|  |  | **HR** | **95%CI** | ***p*** | **HR** | **95%CI** | ***p*** |
| Age | SCU | 1.01 | 0.98–1.04 | 0.30 |  |  |  |
| Female | yes vs. no | 0.38 | 0.18–0.79 | 0.010 | 0.41 | 0.20–0.84 | 0.014 |
| BMI, Kg/m^2^ | SCU | 0.98 | 0.91–1.05 | 0.70 |  |  |  |
| Hypertension | yes vs. no | 1.78 | 0.83–3.82 | 0.13 |  |  |  |
| Hyperlipidemia | yes vs. no | 2.00 | 1.01–3.95 | 0.044 |  |  |  |
| Current smoking | yes vs. no | 0.90 | 0.60–1.34 | 0.61 |  |  |  |
| Diabetes mellitus | yes vs. no | 0.74 | 0.36–1.51 | 0.42 |  |  |  |
| Prior MI | yes vs. no | 1.90 | 1.01–3.60 | 0.047 |  |  |  |
| Prior PCI | yes vs. no | 1.62 | 0.87–3.01 | 0.12 |  |  |  |
| Prior CVA | yes vs. no | 3.33 | 0.95–11.5 | 0.059 |  |  |  |
| Peripheral artery disease | yes vs. no | 1.23 | 0.63–2.35 | 0.53 |  |  |  |
| COPD | yes vs. no | 3.13 | 0.89–10.93 | 0.073 |  |  |  |
| CKD | yes vs. no | 2.19 | 1.14–4.18 | 0.018 | 2.15 | 1.16–3.96 | 0.014 |
| ACS | yes vs. no | 2.79 | 1.54–5.04 | 0.001 | 2.75 | 1.55–4.86 | 0.0001 |
| LVEF | SCU | 0.96 | 0.94–0.99 | 0.021 |  |  |  |
| Multivessel disease | yes vs. no | 2.37 | 1.18–4.73 | 0.015 |  |  |  |
| Aspirin | yes vs. no | 1.00 | 0.75–1.51 | 0.653 |  |  |  |
| P2Y12 inhibitors | yes vs. no | 0.93 | 0.44–1.55 | 0.934 |  |  |  |
| Oral anticoagulants | yes vs. no | 1.23 | 0.76–3.01 | 0.354 |  |  |  |
| ACE inhibitors or ARB | yes vs. no | 1.00 | 0.70–1.59 | 0.365 |  |  |  |
| Beta blockers | yes vs. no | 0.94 | 0.63–2.36 | 0.827 |  |  |  |
| Statin | yes vs. no | 0.85 | 0.44–1.99 | 0.413 |  |  |  |
| Ezetimibe | yes vs. no | 0.99 | 0.75–1.52 | 0.765 |  |  |  |
| Territory | LM/LAD vs. other | 1.12 | 0.68–1.82 | 0.64 |  |  |  |
| Type of lesion | De Novo vs. other | 0.41 | 0.18–0.88 | 0.023 | 0.37 | 0.17–0.80 | 0.012 |
| Serial lesions | yes vs. no | 0.80 | 0.38–1.69 | 0.57 |  |  |  |
| Location | proximal vs. other | 1.56 | 0.92–2.65 | 0.095 |  |  |  |
| AHA/ACC classification | A/B1 vs. B2/C | 0.60 | 0.34–1.05 | 0.077 |  |  |  |
| Severe calcifications | yes vs. no | 0.92 | 0.38–2.26 | 0.86 |  |  |  |
| Severe tortuosity | yes vs. no | 0.32 | 0.06–1.72 | 0.18 |  |  |  |
| RVD | SCU | 1.02 | 0.69–1.51 | 0.89 |  |  |  |
| Diameter stenosis | SCU | 1.00 | 0.97–1.03 | 0.58 |  |  |  |
| Lesion length | SCU | 0.98 | 0.95–1.01 | 0.22 |  |  |  |
| MLD | SCU | 1.03 | 0.89–1.18 | 0.66 |  |  |  |
| FFR | SCU | 0.02 | 0.0007–0.74 | 0.034 |  |  |  |
| FFR–guided | yes vs. no | 0.04 | 0.0002–8.97 | 0.25 |  |  |  |

SCU, single change unit; BMI, body mass index; MI, myocardial infarction; PCI, percutaneous coronary intervention; CVA, cerebrovascular accident; COPD, chronic obstructive pulmonary disease; CKD, chronic kidney disease; ACS, acute coronary syndrome; LVEF, left ventricular ejection fraction; ACE, angiotensin converting enzyme; ARB, angiotensin 2 receptor blocker; AHA, American Heart Association; ACC, American College of Cardiology; RVD, reference vessel diameter; FFR, fractional flow reserve.

Supplemental Table 6. Univariate and multivariable analyses for TVF in study patients.

|  |  | **Univariate** | | | **Multivariable** | | |
| --- | --- | --- | --- | --- | --- | --- | --- |
|  |  | **HR** | **95%CI** | ***p*** | **HR** | **95%CI** | ***p*** |
| Age | SCU | 1.0 | 0.98–1.02 | 0.74 |  |  |  |
| Female | yes vs. no | 0.42 | 0.23–0.75 | 0.003 | 0.51 | 0.28–0.91 | 0.024 |
| BMI, Kg/m^2^ | SCU | 0.97 | 0.92–1.04 | 0.49 |  |  |  |
| Hypertension | yes vs. no | 1.41 | 0.77–2.56 | 0.25 |  |  |  |
| Hyperlipidemia | yes vs. no | 0.97 | 0.59–1.57 | 0.90 |  |  |  |
| Current smoking | yes vs. no | 1.10 | 0.82–1.47 | 0.50 |  |  |  |
| Diabetes mellitus | yes vs. no | 0.55 | 0.29–1.02 | 0.05 | 0.49 | 0.26–0.91 | 0.024 |
| Prior MI | yes vs. no | 0.85 | 0.49–1.46 | 0.56 |  |  |  |
| Prior PCI | yes vs. no | 0.71 | 0.42–1.18 | 0.18 |  |  |  |
| Prior CVA | yes vs. no | 1.25 | 0.46–3.44 | 0.65 |  |  |  |
| Peripheral artery disease | yes vs. no | 0.97 | 0.59–1.60 | 0.93 |  |  |  |
| COPD | yes vs. no | 1.17 | 0.47–2.91 | 0.72 |  |  |  |
| CKD | yes vs. no | 1.30 | 0.81–2.08 | 0.26 |  |  |  |
| ACS | yes vs. no | 2.88 | 1.78–4.67 | 0.000 |  |  |  |
| LVEF | SCU | 0.97 | 0.95–0.99 | 0.004 | 0.96 | 0.94–0.99 | 0.004 |
| Multivessel disease | yes vs. no | 4.95 | 2.47–9.93 | 0.000 | 4.71 | 2.37–9.59 | 0.000 |
| Aspirin | yes vs. no | 0.94 | 0.70–1.53 | 0.731 |  |  |  |
| P2Y12 inhibitors | yes vs. no | 0.98 | 0.40–1.59 | 0.874 |  |  |  |
| Oral anticoagulants | yes vs. no | 1.11 | 0.79–2.97 | 0.453 |  |  |  |
| ACE inhibitors or ARB | yes vs. no | 1.03 | 0.82–1.43 | 0.378 |  |  |  |
| Beta blockers | yes vs. no | 0.90 | 0.59–2.01 | 0.723 |  |  |  |
| Statin | yes vs. no | 0.83 | 0.43–1.95 | 0.325 |  |  |  |
| Ezetimibe | yes vs. no | 0.95 | 0.73–1.43 | 0.632 |  |  |  |
| Negative_FFR | yes vs. no | 0.64 | 0.46–0.88 | 0.007 |  |  |  |

SCU, single change unit; BMI, body mass index; MI, myocardial infarction; PCI, percutaneous coronary intervention; CVA, cerebrovascular accident; COPD, chronic obstructive pulmonary disease; CKD, chronic kidney disease; ACS, acute coronary syndrome; LVEF, left ventricular ejection fraction; ACE, angiotensin converting enzyme; ARB, angiotensin 2 receptor blocker; AHA, American Heart Association; ACC, American College of Cardiology; RVD, reference vessel diameter; FFR, fractional flow reserve.


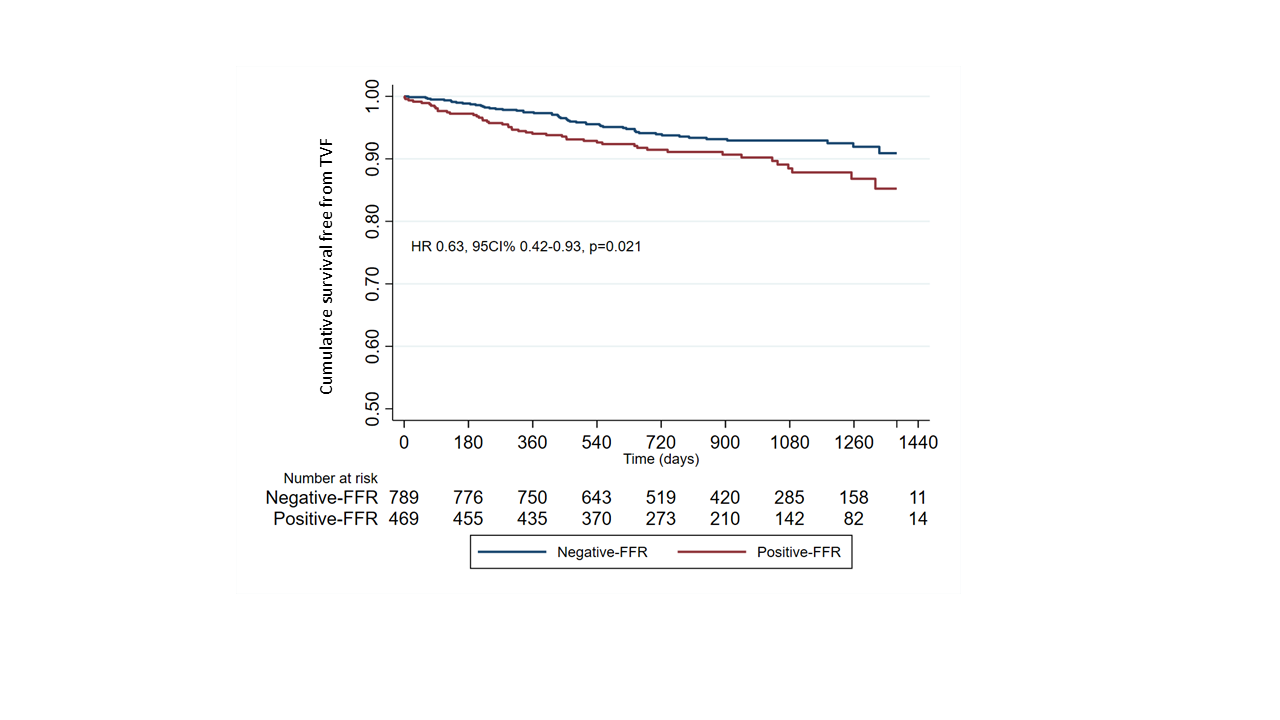


Supplemental Fig. 1. Cumulative free survival from TVF in negative-FFR and positive-FFR patients.

FFR, fractional flow reserve; HR, hazard risk





Supplemental Fig. 2. Subgroup analysis after stratification between negative- and postive-FFR patients.

HR, hazard risk; CKD, chronic kidney disease; CCS, chronic coronary syndrome; ACS, acute coronary syndrome; LVEF, left ventricular ejection fraction; FFR, fractional flow reserve.
